# Effects of Chicken Interferon Gamma on Newcastle Disease Virus Vaccine Immunogenicity

**DOI:** 10.1371/journal.pone.0159153

**Published:** 2016-07-13

**Authors:** Stivalis Cardenas-Garcia, Robert P. Dunwoody, Valerie Marcano, Diego G. Diel, Robert J. Williams, Robert M. Gogal, Corrie C. Brown, Patti J. Miller, Claudio L. Afonso

**Affiliations:** 1 Southeast Poultry Research Laboratory, Agricultural Research Service, United States Department of Agriculture, Athens, Georgia, United States of America; 2 Department of Veterinary Pathology, College of Veterinary Medicine, The University of Georgia, Athens, Georgia, United States of America; 3 School of Medicine, Memorial University Medical Center, Mercer University, Savannah, Georgia, United States of America; 4 Department of Veterinary and Biomedical Sciences, South Dakota State University, Brookings, South Dakota, United States of America; 5 Department of Biosciences and Diagnostic Imaging, College of Veterinary Medicine, The University of Georgia, College of Veterinary Medicine. Athens, Georgia, United States of America; Thomas Jefferson University, UNITED STATES

## Abstract

More effective vaccines are needed to control avian diseases. The use of chicken interferon gamma (chIFNγ) during vaccination is a potentially important but controversial approach that may improve the immune response to antigens. In the present study, three different systems to co-deliver chIFNγ with Newcastle disease virus (NDV) antigens were evaluated for their ability to enhance the avian immune response and their protective capacity upon challenge with virulent NDV. These systems consisted of: 1) a DNA vaccine expressing the Newcastle disease virus fusion (F) protein co-administered with a vector expressing the chIFNγ gene for *in ovo* and booster vaccination, 2) a recombinant Newcastle disease virus expressing the chIFNγ gene (rZJ1*L/IFNγ) used as a live vaccine delivered *in ovo* and into juvenile chickens, and 3) the same rZJ1*L/IFNγ virus used as an inactivated vaccine for juvenile chickens. Co-administration of chIFNγ with a DNA vaccine expressing the F protein resulted in higher levels of morbidity and mortality, and higher amounts of virulent virus shed after challenge when compared to the group that did not receive chIFNγ. The live vaccine system co-delivering chIFNγ did not enhanced post-vaccination antibody response, nor improved survival after hatch, when administered *in ovo*, and did not affect survival after challenge when administered to juvenile chickens. The low dose of the inactivated vaccine co-delivering active chIFNγ induced lower antibody titers than the groups that did not receive the cytokine. The high dose of this vaccine did not increase the antibody titers or antigen-specific memory response, and did not reduce the amount of challenge virus shed or mortality after challenge. In summary, regardless of the delivery system, chIFNγ, when administered simultaneously with the vaccine antigen, did not enhance Newcastle disease virus vaccine immunogenicity.

## Introduction

There is a need for more reliable avian vaccines that prevent infection, replication and shedding of respiratory viruses. Several attempts have been made to develop improved vaccines, including the development of vaccines homologous to virulent NDV (vNDV) strains (which reduce vNDV shedding more efficiently than the standard LaSota vaccine [1–3]), but the effectiveness of these homologous vaccines is still insufficient to prevent completely the shedding of challenge vNDV strains.

Newcastle disease (ND), caused by infections of poultry species with vNDV, remains a significant problem for poultry production worldwide. Vaccination alone prevents disease under experimental conditions, however, it does not eliminate the occurrence of outbreaks with vNDV in countries where the disease is endemic, nor prevents replication of challenge viruses [4–11]. Live-attenuated and inactivated vaccines are the primary vaccines used in ND prevention and control strategies around the world. These vaccines are usually administered by ocular-nasal instillation and replicate in the mucosa stimulating effective cell-mediated (CMI) and antibody-mediated (AMI) immune responses. Live vaccines (i.e. LaSota) may cause a mild respiratory disease affecting productivity [12], whereas inactivated vaccines, while not posing the risk of adverse vaccine reactions, induce only humoral antibody responses with larger amounts of vNDV shed after infection compared to the live NDV vaccines [13].

Chicken IFNγ is a cytokine with pleotropic functions and with multiple similarities to its orthologue in mammals. It contains 169 amino acids (aa) including a 19-aa-signaling segment; the secreted protein contains 145 aa with a molecular weight of approximately 16.8 kDa [14]. It is primarily secreted by T lymphocytes [15] and NK cells. It is the major modulator of macrophage activation in birds [14, 16, 17], it is capable of inhibiting viral replication [18, 19], promotes development of the Th1 response by inhibiting Th2 cytokine production (IL-4 and IL-10) [20, 21], promotes expression of MHC I [22] and MHC II [16, 22], and it enhances antigen presentation and antigen processing and destruction of intracellular pathogens [21]. Interferon gamma has been identified in other bird species such as: duck [19], goose [23], turkey [24], pigeon [25], pheasant, quail and Guinea fowl [24], which indicates that it represents a natural component of the avian immune system. As with other chicken cytokines, chIFNγ signaling pathways is not well understood, but it is presumed to follow the classical JAK-STAT signaling pathway as the mammalian cytokines [21, 26, 27].

The use of IFNγ as a vaccine adjuvant may improve vaccine efficacy. Studies performed in mammals have revealed an immunomodulatory effect of IFNγ as a vaccine adjuvant. For example, increased survival of immunocompromised mice vaccinated with a combination of IFNγ plus malaria antigens was reported upon challenge with malaria [28]. Additionally, IFNγ was able to increase antigen-specific antibody responses to hepatitis B virus and the HIV gp120 protein, increasing antigen-specific T cell proliferative response [29, 30]. Improved protection and enhanced immune responses in avian species have also been reported. Lowenthal and collaborators showed that chIFNγ was able to enhance the antigen-specific AMI response in chickens when co-administered with sheep red blood cells (SRBCs). The maximum effect of chIFNγ was observed 4 to 6 weeks after vaccination and required a high amount of purified protein (10 μg) inoculated intraperitoneally [31]. In addition, increased antibody and cellular responses, and improved overall protection against vNDV challenge have been previously reported after co-administration of chIFNγ with DNA and recombinant fowl pox virus vaccines in chickens and turkeys [32–34]. Chicken IFNγ also improved protection against other avian pathogens such as *Eimmeria tenella*, *Eimmeria acervulina*, chicken anemia virus and Marek’s disease virus [35–40]. However, no commercial product has ever reached the market, and some controversial results ([36, 37, 41, 42]) highlight the need to conduct further evaluation on the utility of this protein as an effective adjuvant for vaccines.

The use of chIFNγ as vaccine adjuvant implies the development of an adequate system to produce and deliver the cytokine in a reliable and economic manner. Chicken IFNγ use as a vaccine adjuvant in poultry would require massive production at low cost in order to supply the billions of doses needed every year. We have developed systems for production and delivery of chIFNγ that can reduce the costs and facilitate administration. A DNA vaccine expressing the viral antigen along with chIFNγ in a manner that can be easily applied *in ovo* was evaluated. A recombinant NDV expressing chIFNγ simultaneously with the antigen during the course of NDV replication was developed and evaluated in eggs and chickens. Both systems have the advantage of not requiring cytokine purification, which make them suitable delivery systems to fulfill the low cost demands of the poultry industry.

In the present study, we characterized three different systems to deliver chIFNγ during vaccination with NDV, in order to study its potential immunomodulatory effects on NDV vaccines. These systems consisted of: 1) a DNA vaccine plasmid expressing the NDV F protein and chIFNγ; 2) a recombinant NDV expressing chIFNγ (used as live delivery vector), and 3) the same recombinant NDV-chIFNγ system utilized as an inactivated vaccine. The effects of chIFNγ on viral shedding, morbidity and mortality were evaluated. Based on previous reports, we initially hypothesized that these three vaccination systems delivering chIFNγ would improve CMI and AMI responses, as well as the overall protection after challenge with vNDV. However, our results showed that co-delivering chIFNγ with antigen using three vaccination systems, under the parameters described here, did not improve the immunogenicity or the protective efficacy of the evaluated vaccine candidates.

## Materials and Methods

### Viruses

Virulent NDV ZJ1 (vZJ1) (Goose/China/ZJ1/2000; GB AF431744.3) was used as a challenge virus in the vaccination experiments. NDV strain LaSota (LS) is used worldwide as a live or inactivated vaccine and thus, served as a control vaccine in our immunization-challenge experiments. Recombinant ZJ1*L (rZJ1*L) is an attenuated version of vZJ1 that was previously generated in our laboratory through reverse genetics; this virus was also included as a control vaccine virus for all the characterization and immunization experiments reported in the present study. All three viruses were obtained from the Southeast Poultry Research Laboratory (SEPRL, USDA-ARS, Athens, GA) viral stocks or repository, and were propagated in 10-day-old specific-pathogen-free (SPF) embryonated chicken eggs (ECEs). The recombinant modified vaccinia virus Ankara expressing the T7 RNA polymerase (MVA/T7) (a gift from Bernard Moss, National Institute of Health) was propagated in primary chicken embryo fibroblast cells (CEF) and was used to rescue the recombinant viruses.

### Chickens, eggs and cells

Southeast Poultry Research Laboratory White Leghorn SPF flocks were the source of all 10-day-old ECEs, 2- and 4-week-old chickens used in every characterization and immunization-challenge experiment. Birds were housed in brooder cages or negative pressure isolators in a biosecurity level 2 enhanced animal (ABSL-2E) facility at vaccination, and transferred into negative pressure isolators in an ABSL-3E facility to be challenged with vZJ1. Birds were provided with food and water *ad libitum*.

Hep-2 cells (ATCC® CCL-23™) and DF-1 cells (ATCC® CRL-12203™) were grown and maintained in high glucose Dulbecco’s modified Eagle’s media (DMEM) supplemented with 10% fetal bovine serum (FBS), 100 U/mL of Penicillin and 100 μg/mL of Streptomycin, and incubated at 37°C under 5% CO_2_ atmosphere. These cell lines were used for virus rescue procedures and protein expression assays, respectively. HD11 cells, a chicken macrophage-like cell line, were grown and maintained in RPMI 1640 media supplemented with 10% FBS, 2 mM L-Glutamine, 100 U/mL of Penicillin and 100 μg/mL of Streptomycin, and incubated at 37°C under 5% CO_2_ atmosphere. This cell line was used for the IFNγ bio-activity assay.

### Development and characterization of plasmids expressing NDV F and chIFNγ genes

The F gene was amplified by PCR from NDV ZJ1 cDNA using Phusion polymerase (New England Biolabs). The amplicons were then digested with Nco I and Not I restriction enzymes and further ligated into the cloning site of the Novagen pTriEx-3 expression vector (cat# 70823; Millipore, Billerica, MA) digested with the same restriction enzymes. The expression plasmid containing the F gene was named pTriEX-ZJ1F. The recombinant plasmid containing chINFγ was generated from the plasmid pCRINFγ (mentioned above) by transferring the chINFγ gene. The resulting plasmid was named pTriEX-INFγ. The recombinant expression plasmids were transformed into Nova Blue E. coli (Millipore) following manufacturer’s instructions. Thereafter, single colonies were grown overnight in LB broth supplemented with 100 μg/mL of ampicillin and purified using the Endotoxin Free plasmid Giga Prep Kit (Cat. # 12391; Qiagen) following manufacturer’s protocol, for their use in protein expression and vaccination experiments.

#### chINFγ production

Protein production was determined by western blotting. Briefly, pTriEX vector, pTriEX-ZJ1-F and pTriEX-IFNγ were individually transfected into DF-1 cells using Lipofectamine 2000 or Lipofectamine LTX (Invitrogen) by the manufacturer’s protocol for transient expression. Cell monolayers were detached by incubation in media with 0.05% Trypsin with EDTA (Gibco). Cells were pelleted to remove media and then re-suspended in 1X phosphate buffered saline (PBS) mixed with SIGMAFAST protease inhibitor cocktail tablets, EDTA free (Sigma-Aldrich, San Luis, MO). Cells were lysed by multiple freeze-thaw cycles. Insoluble material was pelleted at 12000 x g for 10 min at 4°C. Supernatants were stored at -80°C for later analysis. Relative protein concentrations of cell lysates were determined with a BCA protein assay (Pierce). All samples were diluted with 1X PBS with protease inhibitors to match the protein concentration of the most dilute sample. Cell lysates were boiled for 5 min after addition of 2X laemmLi buffer with 350 mM dithiothreitol (1:1) and analyzed through western blotting using Mini-PROTEAN TGX gels (Bio-Rad), and anti-NDV-F-gene and anti-chIFNγ (KingFisher Biotech) antibodies.

### Development and characterization of rZJ1*L/IFNγ

#### Construction of recombinant cDNA full length clone ZJ1*L/IFNγ

Plasmid pNDV/ZJ1 used as back bone to construct our cDNA full length clone expressing chIFNγ, was kindly donated by Dr. Lui and collaborators from the Animal Infectious Disease Laboratory, School of Veterinary Medicine, Yangzhou University, Yangzhou, PR China. This plasmid contains the whole genomic cDNA of the wild type vNDV ZJ1 and its development and characterization have been previously described [43]. The plasmid called pCRIFNγ containing the chIFNγ gene with gene start and gene end (GS and GE, respectively) codons was previously developed in our laboratory [44] and was used as the source for chIFNγ gene to be inserted into the ZJ1 genome. Development of the full length cDNA was conducted as described by Susta and collaborators [44] with a few modifications to the protocol. Briefly, the Fusion (F) protein cleavage site from pNDV/ZJ1 was attenuated through site directed mutagenesis using the Phusion Site-Directed Mutagenesis kit (New England Biolabs Inc., Ipswich, MA) according to the manufacturer’s instructions, giving origin to pNDV/ZJ1*L. Thereafter, to insert the chIFNγ gene into the ZJ1 backbone, the 2857–5637 region of the ZJ1 genome was amplified from pNDV/ZJ1, and cloned into the pCR2.1 vector (Invitrogen, Carlsbad, CA). This region was sub-cloned into the pUC19 vector (Invitrogen) using HindIII and XbaI restriction enzymes, resulting in the plasmid pUCZJ1. The chIFNγ gene was then transferred from the pCRIFNγ plasmid into the pUCZJ1 plasmid through the ApaI restriction site, and the resulting intermediate plasmid was named pUCZJ1-IFNγ. Plasmid pUCZJ1-IFNγ was then digested with AgeI/PsiI restriction enzymes, and the region containing the chIFNγ with GS, GE and ApaI restriction sites was sub-cloned into the full-length pNDV/ZJ1*L between the P and M genes of the ZJ1 genome, within the untranslated regions (UTRs) of the P gene. The resultant plasmid was designated pNDV/ZJ1*L-IFNγ.

#### Virus rescue

The recombinant virus was rescued by reverse genetic techniques from pNDV/ZJ1*L- IFNγ as described elsewhere [1], using Hep-2 cells grown and maintained in Dulbeco’s Modified Eagle Medium (DMEM) (Corning cellgro, Invitrogen), supplemented with 5% Fetal Bovine Serum (FBS) and antibiotics (100 U/mL penicillin and 100 μg/mL streptomycin), at 37°C with a 5% CO2 atmosphere. The rescued virus was designated as rZJ1*L/IFNγ and further subjected to RNA extraction, RT-PCR and sequencing to confirm its identity.

#### Intracerebral pathogenicity index (ICPI)

One day-old SPF White Leghorn chickens were inoculated intracerebrally with 50 μL of a 1:10 dilution of allantoic fluid (AF) harvested from ECEs infected with either vZJ1, LS, rZJ1*L and rZJ1*L/IFNγ. Birds were monitored every 24 hrs during 8 days and scored as follows: 0 = normal, 1 = sick or 2 = dead. Any virus with an ICPI ≥ 0.7 was considered virulent NDV [45, 46].

#### Mean death time (MDT) and virus titration in eggs

Ten day-old SPF ECEs were inoculated as preciously described [45, 46] with vZJ1, LS, rZJ1*L or rZJ1*L/IFNγ. The MDT was expressed as the mean time in hours at which the highest dilution killed 100% of the embryos. Allantoic fluids were harvested after death or at the end of the experimental period (7 days post-inoculation) from chilled eggs and used to determine virus titers by HA test and using the Spearmann-Karber method to calculate the EID50/mL [47].

#### Expression of chIFNγ from DF-1 cells and in ECEs infected with live virus

DF-1 cells were maintained in DMEM supplemented with 10% FBS, 100 U/mL of penicillin and 100 mg/mL of streptomycin, at 37°C with a 5% CO2 atmosphere. Cells were seeded in 6-well plates at a density of 1x10^6^ cells/well and incubated overnight. Cells were washed with 1X PBS three times and 500 μL of inoculum containing either rZJ1*L (10 MOI) or rZJ1*L/chIFNγ (10 MOI) were added to the designated wells in triplicates. Inoculated cells were incubated at 37°C with a 5% CO2 atmosphere for 1 hr, rocking the plates every 15 min. The inoculum was removed from each well and fresh DMEM supplemented with 10% FBS, 100 U/mL of penicillin and 100 mg/mL of streptomycin was added. Twenty four hours post infection the cell layers and supernatants were mixed with 2X laemmLi buffer, boiled for 5 min at 100°C, and stored at -80°C until processed. Cell lysates and supernatants were analyzed through western blotting using 8–16% polyacrylamide gels and an anti-chIFNγ polyclonal antibody (Cat.# PB0442C-100, KingFisher Biotech, Inc., Saint Paul, MN). In addition, 10-day-old ECEs were inoculated with ZJ1*L or rZJ1*L/IFNγ at an EID_50_/egg of 10^3^. Allantoic fluids were collected 24, 48, 72 and 96 hrs post inoculation (3 eggs per time point, per virus) and analyzed through ELISA using a commercial antibody pair to detect chIFNγ (Cat.# CAC1233, Invitrogen). Concentrations of chIFNγ were also determined by ELISA from vaccine virus stocks (ZJ1*L and ZJ1*L/IFNγ) and uninfected AF treated with BPL; these BPL-treated AFs were used to prepare the emulsified inactivated vaccines.

#### Determination of chIFNγ bio-activity from BPL-treated AFs

In order to confirm bio-activity of the *chIFNγ* present in AF infected with ZJ1*L/IFNγ after inactivation with β-Propiolactone (BPL), HD11 cells were stimulated with various BPL-treated AFs to determine macrophage activation through quantification of nitrites (a sub-product of nitric oxide). The day prior to the assay, cells were seeded at a density of 4x10^5^ cells/well in a 96-well plate and incubated overnight. Thereafter, the media was replaced with 100 μL of supplemented RPMI 1640 without phenol red per well. Then, 100 μL of a 1:10 dilution of either BPL-treated uninfected AF (BPL-AF), BPL-inactivated ZJ1*L (BPL-ZJ1*L) or BPL-inactivated ZJ1*L/IFNγ (BPL-ZJ1*L/IFNγ) were added per well in triplicates and incubated at 37°C under 5% CO_2_ atmosphere. Forty eight hours post-stimulation, 50 μL of each replicate per treatment were tested for nitrite concentration in duplicate using the Griess Reagent System (Cat.# G2930, Promega; Madison, WI).

### Immunization and challenge experiments

#### *In ovo* immunization with DNA vaccines

Vaccines were prepared by diluting the recombinant plasmids in TE buffer. One vaccine dose contained a total 150 μg of plasmid DNA in 200 μL of TE buffer except for the control group which contained 200 μL of TE buffer alone. Eighteen-day-old SPF ECEs were split into seven groups (n = 30 eggs/group). Every egg was inoculated by amniotic sac route with one dose of the corresponding DNA vaccine (TE buffer, pTriEX, pTriEX-ZJ1-F or pTriEX-ZJ1-F + pTriEX-IFNγ) (Table 1). Two weeks after hatch, birds were boosted intramuscularly (in the right pectoral muscle) with the corresponding vaccine using the same dose. Hatchability and survival after vaccination were evaluated. Two weeks after booster vaccination, birds were transferred into an ABSL-3 facility and challenged with vZJ1 (10^5^ EID_50_/bird) by ocular and choanal instillation. Birds were monitored for 14 days post-challenge (dpc) for characteristic NDV clinical sings and mortality. Oropharyngeal and cloacal swab samples were collected at day 3 pc to measure challenge virus shedding. Serum samples were collected on day 14 pc to evaluate serological responses.

**Table 1:** DNA-vaccine groups and plasmid combinations.

Groups	Plasmids combinations
pTriEX-ZJ1F	100 μg pTri-ZJ1F plus 50 μg empty plasmid
pTriEX-ZJ1F + pTri-INFγ	100 μg pTri-ZJ1F plus 50 μg pTri-INFγ plasmid
pTriEx	150 μg empty vector plasmid
Sham-vaccinated	TE buffer, no plasmid DNA

#### *In ovo* immunization with live recombinant vaccines

Nineteen-day old SPF ECEs were randomly assigned to either one of 4 vaccine groups and inoculated with brain heart infusion (BHI) (Sham-vaccinated), LS, rZJ1*L or rZJ1*L/IFNγ at a dose of 10^3.5^ EID_50_/egg. Regardless of the treatment, eggs were manually inoculated with 100 μL of the corresponding vaccine or uninfected inoculum through the amniotic route, using 1 cc syringes with 24 G x 1/2”. After vaccination, each group of vaccinated eggs was placed in a 2362E Turbofan Hova-Bator Incubator (by GQF). Each incubator was placed inside of a BSL2 isolator and the temperature and humidity were monitored until 21 days of embryonation. After hatch, chicks were monitored daily for survival and clinical signs until 14 days post-hatch (dph). At 14 dph, 12 chickens from each group were individually identified and serum was collected for serology. These birds were challenged with 10^4.9^ EID_50_/bird of vZJ1 by the ocular and choanal cleft instillation (50 μL each route). Challenged birds were monitored daily for clinical signs and mortality for two weeks. Pre-challenge and post-challenge antibody titers were determined by hemagglutination inhibition (HI) assay [46].

#### Immunization and challenge of 4-week-old SPF chickens with live recombinant vaccines

In order to study the effect of rZJ1*L/IFNγ on juvenile chickens upon challenge, forty four 4-week-old SPF White Leghorn chickens were vaccinated and challenged 2 weeks after vaccination. Birds were vaccinated with 100 μL of BHI, LS, rZJ1*L or rZJ1*L/IFNγ by ocular and choanal cleft instillation (50 μL each route) at 4 weeks of age. The intended dose for each vaccine was 10^6.5^ EID_50_/bird. Two weeks after vaccination, all birds were challenged with vZJ1 at an EID_50_/bird of 10^6.5^. Mortality was recorded until 14 dpc.

#### Inactivated-vaccine preparation

Allantoic fluid (AF) from uninfected ECEs and from ECEs infected with LS, rZJ1*L or rZJ1*L/IFNγ was titrated in 9-11-days-old ECEs and equilibrated to the same titer (EID_50_/mL). Thereafter, AFs were inactivated with β-propiolactone (BPL) as follows: 0.11% (v/v) of BLP was slowly added to the each AF while rocking. After 5 min, the fluids were transferred into new sterile flaks and incubated for 3.5 hrs at room temperature while rocking. Thereafter, the lids of the flasks were opened to allow air to come inside and the flasks were put at 4°C overnight. The next day, the pH was adjusted to 7 using pH strips and sterile sodium bicarbonate. The BPL treated AFs were used to prepare oil emulsion vaccines by mixing 36 mL of mineral oil (ce6vr or Drakeol 6VR) with 3 mL of Arlacel 80 and 1 mL of Tween 80 into a sterile container. A blender and a sterile metal mixing cup were assembled and the oil mix was poured inside the cup, followed by the corresponding BPL treated AF. The mixture was blended as follows: 1 min low, 1 min rest, 1 min low, 1 min rest and last 30 sec high. The blended vaccines were poured into sterile vaccine bottles and properly sealed. Four emulsified vaccine preparations (Sham, LS, ZJ1*L and ZJ1*L/IFNγ) were kept at 4°C until needed.

#### Immunization with inactivated recombinant vaccines

Two different vaccine doses were evaluated in two independent experiments to study the effect of ZJ1*L/IFNγ on AMI and CMI responses, and post-challenge viral shedding and survival. The first experiment evaluated the immune response and did not have a challenge component. The second experiment did evaluate the immunity to a virulent challenge virus. All vaccines were equilibrated to have the same titer before being inactivated to allow them to be compared. Final titers from equilibrated vaccines, before BPL inactivation, were 10^8.1^ EID_50_/mL and 10^9.1^ EID_50_/mL for the first (no-challenge) and second (with challenge) experiments, respectively. Seventy two 2-week-old SPF White Leghorn chickens were randomLy allocated into four groups (n = 18) and vaccinated subcutaneously (SC) with 300 μL/bird of either sham-vaccine (uninfected AF), LS, ZJ1*L or ZJ1*L/IFNγ emulsions. Three weeks after vaccination, blood was collected from brachial vein without anticoagulant for serology and each bird was boosted SC with 300 μL of the corresponding emulsified vaccine. One week after the booster, blood samples were collected for serology as before. Six birds from each group vaccinated with the 10^9.1^ EID_50_/mL-vaccine batches, were euthanized by cervical dislocation and the spleens were aseptically removed for lymphocyte isolation as described below. The remaining birds (n = 12/group) were transferred to an ABSL-3 facility and challenged with vZJ1 (10^9.5^ EID_50_/bird). Oropharyngeal and cloacal swab samples were collected 2 and 4 days post-challenge (dpc). Birds were monitored daily for up to 14 dpc for clinical signs and mortality. At the end of the experiment (14 dpc), blood was collected from every survivor for serology. Birds vaccinated with 10^8.1^ EID_50_/mL-vaccine batches were terminated after bleeding procedure.

### Evaluation of the recall CMI response in birds immunized with inactivated vaccines

#### Lymphocyte isolation from spleen

Six birds per vaccine group were euthanized by cervical dislocation one week after booster vaccination (4 weeks after initial vaccination). Spleens were aseptically removed and placed into 50 mL conical tubes containing 15 mL of ice-cold 1X PBS (HyClone) for their subsequent transport a BSL-2 laboratory. Each spleen was gently passed through a 70 μm cell strainer (Fisher) into a sterile petri dish containing 6 mL of room temperature (RT) 1X PBS using the barrel of a 10 cc syringe; the strainer was then rinsed of RT 1X PBS to have a final cell suspension volume of approximately 10 mL. The cell suspensions were pipetted up and down a few times and then transferred into a 50 mL conical tube to be centrifuged at 450 x g for 5 min at room temperature. The supernatants were discarded and the cell pellet re-suspended with 6 mL of RT 1X PBS. Then, 3 mL of cell suspension were overlaid onto 3 mL of Histopaque 1.077 (Sigma) in a 15 mL centrifuge tube and centrifuged at 450 x g for 30 min at 18°C. Following centrifugation and using a glass Pasteur pipette, the opaque interface containing the lymphocytes was removed and washed 3 times in 10 mL RT 1X PBS, centrifuging at 450xg for 10 min at 18°C. Following the final wash, the cells were re-suspended in RPMI-1640 (Hi Clone) supplemented with 10% FBS (Gibco), 1X penicillin-streptomycin mix (Gibco) and 2 mM of L-glutamine (Gibco). Cells were enumerated on a cellometer Auto T4 (Nexcelom, Lawrence, MA) and adjusted to 2.5x10^6^ cells/mL in complete media.

#### Lymphocyte proliferation assay

Recall CMI responses were evaluated with a lymphocyte proliferation assay. This assay was previously standardized to identify the best assay conditions namely temperature and incubation time, addition of alamar blue, cell concentration and the amount of antigen to be used (data not shown). Briefly, cells were seeded into round-bottom 96-well plates (cat. # CLS3799-50EA; Corning Inc., Corning, NY) at 2.5x10^5^ cells/well in 100 μL of complete growth media, and stimulated with either 10 μg/mL of Con A (cat. # C5275-5MG; Sigma-Aldrich), inactivated ZJ1*L (EID_50_/0.1 mL of 10^6.6^, before BPL inactivation) or media only, adding 100 μL/well of each treatment to the to the corresponding wells, in triplicate. Cells were incubate at 41°C in a 5% CO2 environment during 86 hrs and then, 20 μL of alamar blue were added to each well. Plates were read 120 hrs post-stimulation in a micro-plate reader using wavelengths of 570 nm and 600 nm. The calculations were made as described somewhere else (19). Briefly, the readings at 600 nm were subtracted from the readings at 570 nm; subsequently, triplicate readings were averaged and the mean OD per treatment per vaccine group was calculated.

#### Determination of lymphocyte subpopulations in spleen from vaccinated birds

Cells (5.0x10^5^/sample) were stained with anti-chicken CD3, CD4, CD8 and IgM antibodies (Southern Biotech, Birmingham, AL) for 30 min at 4°C in the dark. The cells were washed with 1X concentrated phosphate buffered saline (PBS) and centrifuging at 200 x g for 10 min at 4°C. The cells were re-suspended with 100 μL of PBS and fixed with 100 μL of 2% paraformaldehyde. Samples were evaluated on a BD-LSR II flow cytometer measuring 10,000 events per sample. Values were reported as percent expression.

### Statistical analysis

One-way or two-way ANOVA followed by a multiple comparisons Tukey's test were employed, when appropriate, to analyze HI, viral shedding and cell proliferation assay results. Survival curves were analyzed using the Long-Rank test. Morbidity results were evaluated as proportions, using a two-tailed Z test. Statistical difference was considered with a *P<0*.*05* and the significant differences were denoted by different letters.

### Ethics Statement

All experiments were conducted complying with protocols reviewed and approved by the Southeast Poultry Research Laboratory Institutional Biosafety Committee and were conducted with appropriate measures to maintain biosecurity and biosafety, in accordance with the rules and regulations of the United States Department of Agriculture. General care of chickens was provided in accordance with the procedures reviewed and approved by the Southeast Poultry Research Laboratory Institutional Animal Care and Use Committee (IACUC), under animal use proposal numbers FY2014-01 and FY2014-06, and as outlined in the Guide for the Care and Use of Agricultural Animals in Agricultural Research and Teaching. Humane endpoints were observed and utilized over the entire duration of the experimental study. Birds were checked daily, and signs of clinical disease were recorded. Birds that were either unable or unwilling to eat and/or drink were euthanized immediately by cervical dislocation or by the administration of intravenous sodium pentobarbital (100 mg/kg) by people trained and approved by the IACUC. The morbidity and mortality rates were as expected based on previous experiments.

## Results

### DNA *in ovo* vaccine system

#### Identity of pTriEX-NDVF and pTriEX-IFNγ

The identity of the recombinant plasmids expressing F and IFNγ genes was confirmed by DNA sequencing. The full fusion and IFNγ DNA insert plus specific regions of the pTriEX vector were sequenced with gene-specific and vector-specific primers. Sequencing analysis revealed that both the F and IFNγ genes were complete and inserted in the right region and orientation.

#### Expression of F protein

**Fig 1:**
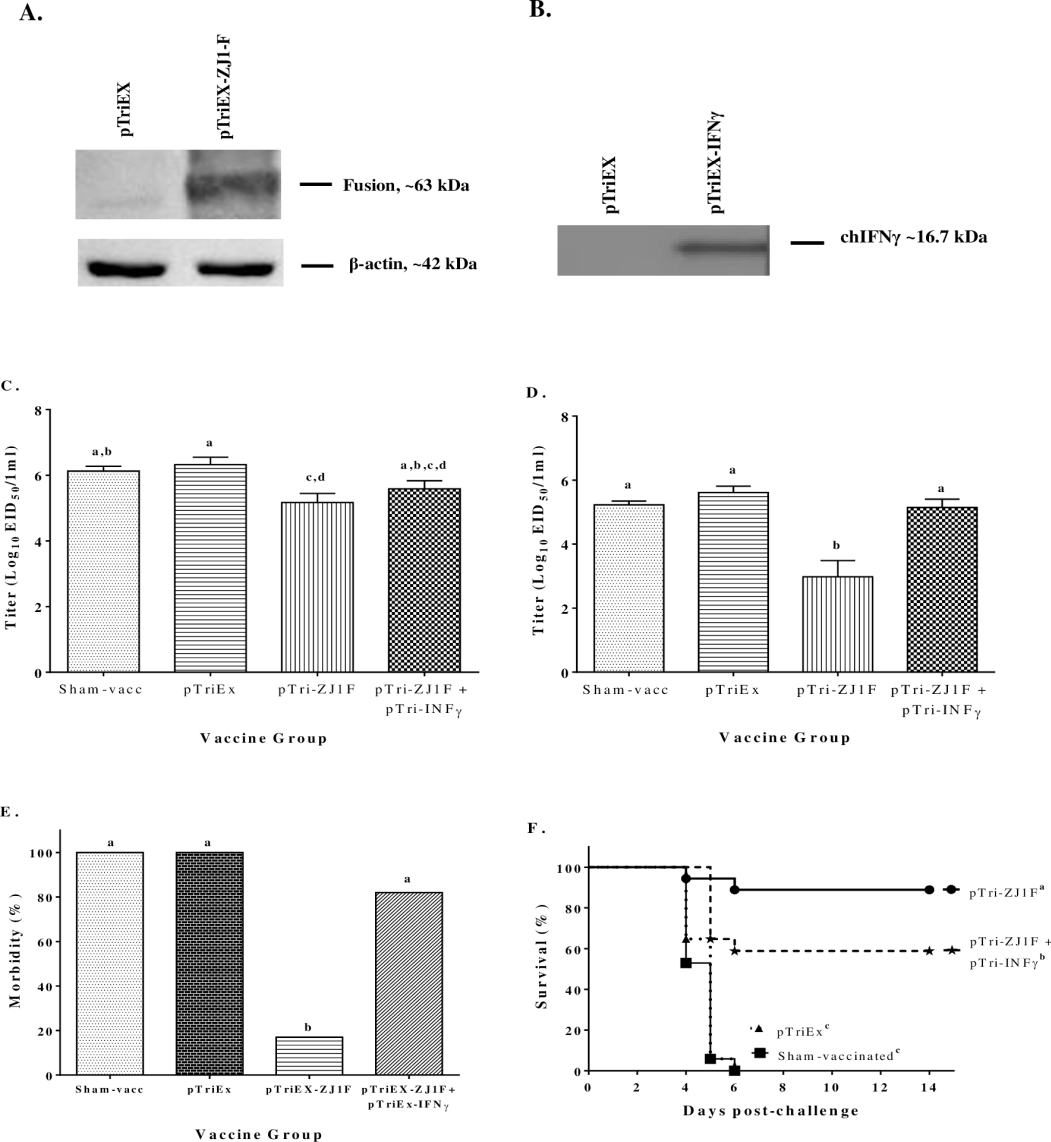
Characterization and evaluation of the effect of co-delivering chIFNγ with a DNA vaccination system. Plasmids expressing NDV F and chIFNγ genes were developed and characterized for their use as DNA vaccines and adjuvant, respectively. DF-1 cells were transfected with pTriEX, pTriEX-ZJ1-F and pTriEX- IFNγ. Cell culture supernatants were tested by western blotting for the presence of F protein **(A)** and chIFNγ **(B)**, respectively. Eighteen-day-old SPF ECEs were inoculated with TE buffer, pTriEX, pTriEX-ZJ1-F, or pTriEX-ZJ1-F plus pTriEX- IFNγ and boosted 2 weeks after hatched. Two weeks after booster vaccination, birds were challenged with vZJ1. Oropharyngeal **(C)** and cloacal **(D)** swab samples were collected 3 days after challenge to measure the amount of challenge virus shed into the environment. Viral titers were determined by quantitative real time reverse transcription polymerase chain reaction (qRRT-PCR). A standard was prepared with a vZJ1 virus stock of know concentration, this was included in every plate and was used to obtain viral titers expressed as EID_50_/mL. Morbidity **(E)** and mortality **(F)** were also evaluated. Viral shedding results were analyzed with One-way ANOVA followed by a multiple comparisons Tukey's test. Differences in morbidity among groups were evaluated using a two-tailed Z test for comparison of sample proportions. Survival curves were analyzed using the Long-Rank test. Statistical difference was considered with a *P<0*.*05*. Significant differences are denoted by different letters.

Results from western blot analysis revealed that pTriEX-NDVF expressed the F protein and while the F protein was also detected from transfected DF-1 cell lysates by western blotting, there was no evidence of the F protein detected from the pTriEX transfected cells (Fig 1A).

#### IFNγ production

The capability of pTriEX-IFNγ to produce chIFNγ was confirmed in vitro. DF-1 cells were transfected with pTriEX or pTriEX-IFNγ. Cell culture supernatants were assayed for the presence of chIFNγ by western blotting, using polyclonal antibodies as described above. Chicken IFNγ was detected in the supernatant of pTriEX-IFNγ transfected DF-1 cells, while there was no protein detected from pTriEX-transfected DF-1 cell (Fig 1B).

#### Effects of chIFNγ delivered through an *in ovo* DNA vaccine system

Delivery of chIFNγ by plasmids during vaccination was evaluated. Eighteen-day-old SPF ECEs were inoculated with plasmid DNA or TE buffer alone, boosted 2 weeks after hatch and challenged with vZJ1 2 weeks after booster vaccination. The effects of chIFNγ on hatchability, challenge virus shedding, morbidity and mortality after challenge were evaluated. Hatchability after *in ovo* vaccination ranged between 90% (Sham-, pTriEX- and pTriEX-ZJ1F+pTriEX-IFNγ-vaccinated groups) and 93% (pTriEX-ZJ1F-vaccinated group) demonstrating no significant differences compared to the control.

Evaluation of viral shedding after challenge did show differences between groups. Vaccination with pTriEX-ZJ1F alone significantly reduced the viral shedding from oropharynx and cloaca compared to the control groups at 3 dpc, and most importantly, it shed less virus than the pTriEX-ZJ1F+pTriEX-IFNγ-vaccinated group (Fig 1C and 1D). Thus, the co-expression of IFNγ with the DNA vaccine pTriEX-ZJ1F led to higher virus shedding when compared to pTriEX-ZJ1F vaccinated group, whereas no differences were observed between Sham- and pTriEX-vaccinated groups (Fig 1C and 1D).

Notably, chIFNγ affected morbidity and mortality induced by challenge with vNDV. Co-administration of pTriEX-IFNγ with pTriEX-ZJ1F also led to significantly higher morbidity (82%) upon challenge than the administration of pTriEX-ZJ1F alone (17%) (*P = 0*.*0001*). Whereas birds immunized with pTriEX-ZJ1F presented lower morbidity than the non-vaccinated groups (100%) (*P<0*.*0001*). Mortality for Sham-, pTriEX-, pTriEX-ZJ1F+pTriEX-IFNγ- and pTriEX-ZJ1F-vaccinated groups were 100%, 100%, 41% and 11%, respectively. Mortality observed in the Sham-, pTriEX- and pTriEX-ZJ1F-vaccinated groups was as expected, while mortality was significantly higher than expected for the pTriEX-ZJ1F+pTriEX-IFNγ-vaccinated group as compared to the pTriEX-ZJ1F (*P = 0*.*049*) (Fig 1E and 1F).

### Live vaccine system

#### Identity and virulence of rZJ1*L/IFNγ

An attenuated, recombinant NDV expressing the chIFNγ gene was generated by reverse genetics. The attenuation of the cleavage site was confirmed by nucleotide sequencing, determination of the ICPI in one day-old chickens, and the MDT in ECEs. Recombinant ZJ1*L/IFNγ had an ICPI compatible with NDV strains of low virulence (0.00) while the parental virulent virus (vZJ1) exhibited a high ICPI (1.83) value. The fusion protein cleavage site for rZJ1*L/IFNγ was confirmed to be identical to the low virulence cleavage site from LS (_112_G R Q G R↓ L_117_). Furthermore, the MDT value for rZJ1*L/IFNγ (>175 hrs) also classified this virus as a NDV of low virulence.

#### IFNγ production by live rZJ1*L/IFNγ

**Fig 2:**
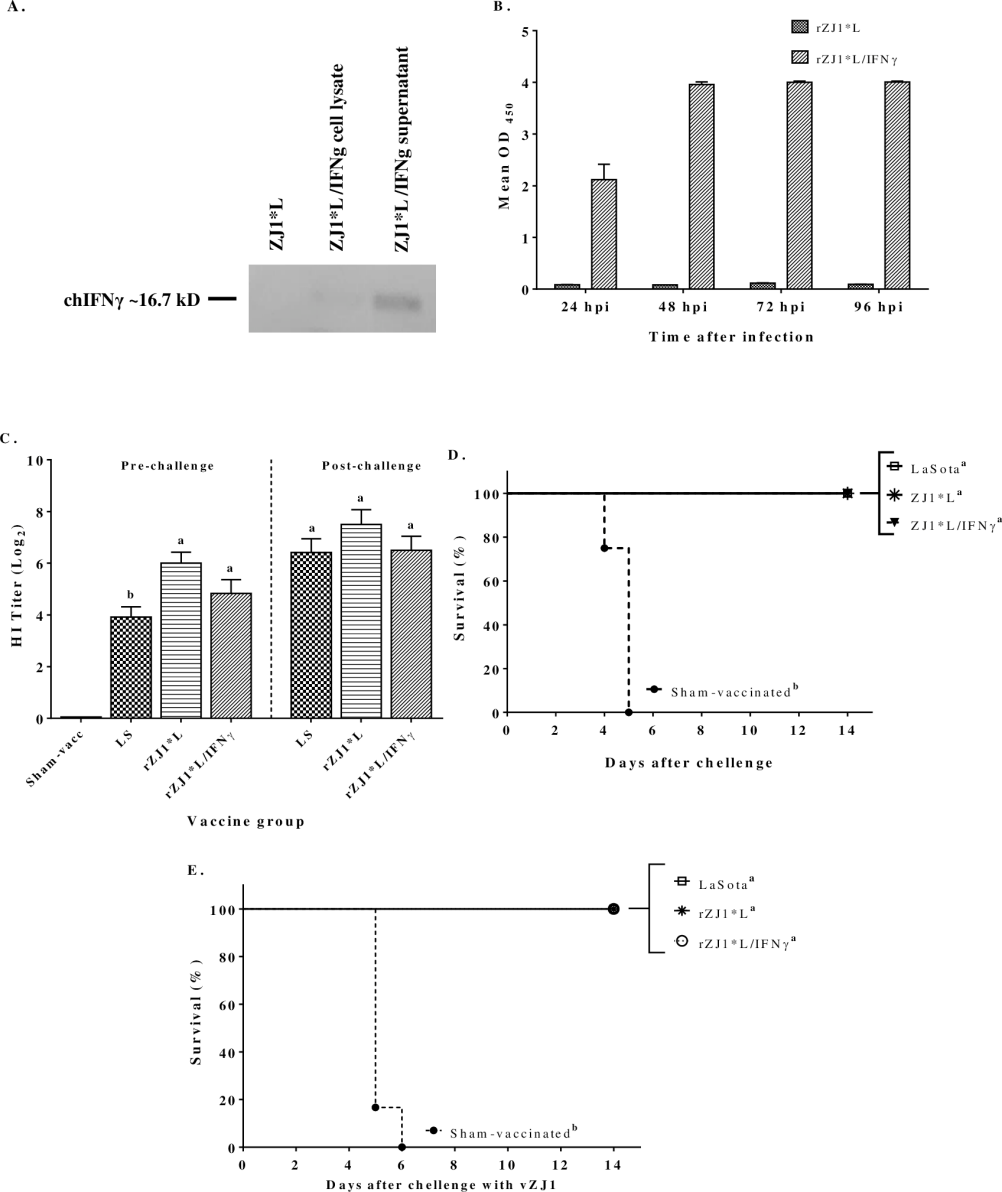
Characterization and evaluation of the effect of co-delivering chIFNγ with a live recombinant vaccination system. DF-1 cells and 10-day-old ECEs **(B)** were infected with live rZJ1*L and rZJ1*L/IFNγ to confirm *in vitro* and *in vivo* expression of chIFNγ. Cell culture supernatants were collected 24 hrs after infection and tested for chIFNγ by western blotting **(A)**. Infected allantoic fluids (AFs) were collected 24, 48, 72, and 96 hrs after infection; chIFNγ concentrations from infected AFs were determined by ELISA **(B).** Nineteen-day-old SPF ECEs were inoculated with BHI, LS, rZJ1*L or rZJ1*L/IFNγ and challenged two weeks after hatch with vZJ1. Sera were collected before and after the challenge for HI antibody titer determination **(C)**. Survival after challenge was recorded **(D)**. Four-week-old SPF chickens were also vaccinated with BHI, LS, rZJ1*L or rZJ1*L/IFNγ and challenged two weeks later with vZJ1 to record mortality **(E)**. HI antibody titers were analyzed with One-way ANOVA followed by a multiple comparisons Tukey's test. Survival curves were analyzed using the Long-Rank test. Statistical difference was considered with a *P<0*.*05*. Significant differences are denoted by different letters.

The ability of rZJ1*L/IFNγ to produce chIFNγ was confirmed *in vivo* and *in vitro*. Ten-day-old SPF ECEs (*in vivo*) and DF-1 cells (*in vitro*) were inoculated with rZJ1*L or rZJ1*L/IFNγ. Cell culture supernatants and cell lysates were assessed for the presence of chIFNγ by western blotting, using polyclonal antibodies. Chicken IFNγ was detected from both DF-1 cell culture supernatants and cell lysates of samples infected with rZJ1*L/IFNγ, while no chIFNγ was detected from rZJ1*L-infected DF-1 supernatants, or from the cell lysates (Fig 2A). In addition, chIFNγ specific ELISA was used to detect production of chIFNγ *in vivo* from infected ECEs. The concentration of IFNγ in rZJ1*L/IFNγ-infected allantoic fluids (AFs) increased over time until reaching peak concentrations between 72 and 96 hrs post-infection that saturated the ELISA. Very low levels of chIFNγ were detected in the rZJ1*L-infected AFs (Fig 2B).

#### Effects of chIFNγ delivered through an *in ovo* live vaccine system

In order to determine the applicability of rZJ1*L/IFNγ as an *in ovo* vaccine and its effect on immune response modulation, 19-day-old SPF ECEs were vaccinated. One of the most important parameters when evaluating *in ovo* vaccines, is the effect of the vaccine on survival after hatching. Here we evaluated the effect of vaccinating with rZJ1*L/IFNγ on the AMI response, on survival after hatch, and on protection by comparing those parameters with standard LS vaccine strain and rZJ1*L. Survival results and hatchability are summarized in Table 2. The greatest post-hatch survival recorded by 14 dph was achieved by the sham-vaccinated control, followed by rZJ1*L and rZJ1*L/IFNγ. The vaccine group with the lowest post-hatch survival rate was LS, as the LS vaccine is expected to cause mortality. According to our results, survival rates increased when ECEs were vaccinated at 19 days of embryonation with both recombinant vaccines (rZJ1*L and rZJ1*L/IFNγ) inducing better survival than LS. However, rZJ1*L/IFNγ vaccine virus expressing chIFNγ induced a lower survival rate compared to the control rZJ1*L vaccine, but the difference was not statistically significant (Table 2). While the pre-challenge antibody titers for the Sham-vaccinated, ZJ1*L and LS groups were significantly different from one another (*P = 0*.*003*), there was no statistical difference in titers between the LS and rZJ1*L/IFNγ groups or between the rZJ1*L and rZJ1*L/IFNγ groups (Fig 2C). There were no statistically significant differences on post-challenge antibody titers, between any of the vaccinated groups (Fig 2C). Although the pre- and post-challenge antibody titers from rZJ1*L/IFNγ group were not statistically different than those for the rZJ1*L group, there were numerical differences that suggest an impairment in antibody response caused when chIFNγ was co-delivered with the vaccine antigen. Despite the differences in the pre-challenge antibody titers between vaccinated groups, no differences in clinical disease or mortality were observed, whereas the sham-vaccinated group reached 100% mortality by 5 days after challenge (Fig 2D). In summary, rZJ1*L/IFNγ administered *in ovo* did not significantly improve survival after hatch or the AMI response when compared to the LS vaccine.(Table 2 and Fig 2C).

**Table 2:** Effect of chIFNγ on hatchability and survival after *in ovo* vaccination with rZJ1*L/IFNγ and challenge with vZJ1.

Vaccine Group	Hatchability (%)	Survival after hatch (%)	Survival after challenge (%)
Sham-vaccinated	92.3^a^	100^a^	0^b^
LS 10^3.5^	92^a^	60.87^b^	100^a^
rZJ1*L 10^3.5^	92.3^a^	80^b^	100^a^
rZJ1*L/IFNγ 10^3.5^	92.5^a^	72.97^b^	100^a^

Hatchability and survival results after hatch and after challenge were analyzed using the Long-Rank test. Statistical difference was considered with a *P<0*.*05*. Statistical differences are denoted by different letter superscripts.

#### Effects of chIFNγ delivered through a live vaccine system in juvenile chicken

The effect of rZJ1*L/IFNγ as live vaccine in juvenile (4-week-old) chickens was also evaluated. Vaccinated chickens were challenged with vZJ1. Survival analysis after challenge showed that 100% of the birds survived regardless of the given vaccine treatment; however, 100% of the sham-vaccinated birds succumbed by day 5 after challenge (Fig 2E).

### Inactivated vaccine system

#### Quantification of chIFNγ from BPL-inactivated rZJ1*L/IFNγ AF

**Fig 3:**
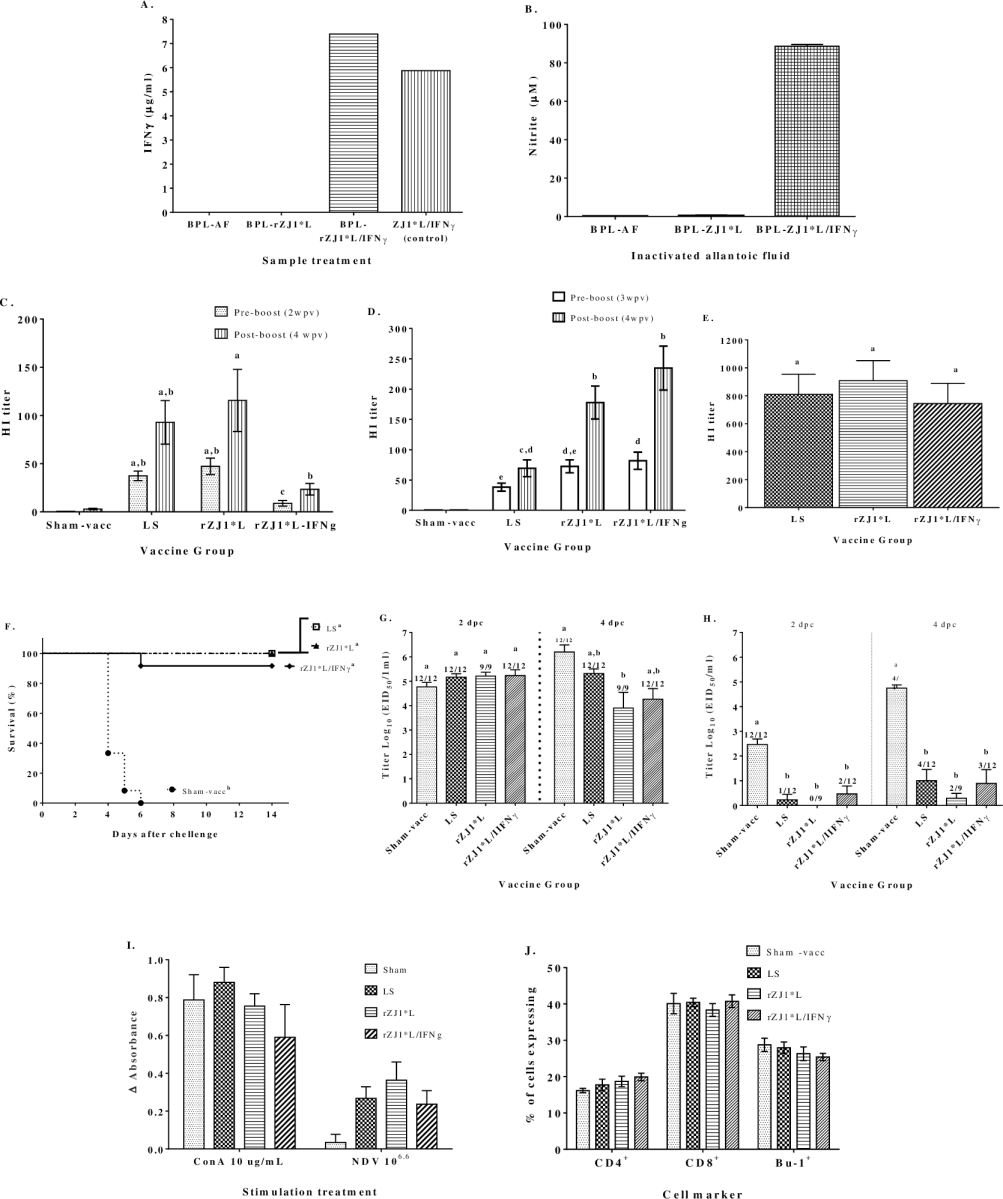
Characterization and evaluation of the effect of co-delivering chIFNγ with an inactivated recombinant vaccination system. Uninfected allantoic fluid (AF), rZJ1*L- and rZJ1*L/IFNγ-infected AFs were inactivated with BPL for inactivated vaccine preparation and tested for chIFNγ concentration by ELISA **(A)**. BPL-treated AFs were used to stimulate HD11 cells and confirm chIFNγ bio-activity through determination of nitrites as a sub-product of nitric oxide induced upon macrophage activation. Stimulated cells were incubated for 48 hrs at 37°C under a 5% CO_2_ atmosphere. Cell culture was used to determine nitrite concentration using the Griess method **(B)**. Two-week-old SPF birds were vaccinated and boosted with inactivated Sham-vaccine, rZJ1*L or rZJ1*L/IFNγ. Two different vaccine doses were tested and serum samples were collected before and after challenge for antibody titer determination by HI test. Pre-challenge HI titers after vaccination with an EID_50_/mL of 10^8.1^ **(C)** and 10^9.1^ **(D)**, and post-challenge titers **(E)** are shown. Mortality was recorded daily for 2 weeks **(F)**. Oropharyngeal **(G)** and cloacal **(H)** swab samples were collected 2 and 4 dpc, viral titers were determined in 10-day-old ECEs and are expressed as EID_50_/mL. One week after booster vaccination, 6 birds from each vaccinated group were euthanized and the spleens were collected for the isolation of lymphocytes to be used in a proliferation assay to measure antigen-specific memory T cell response **(I)** and to determine T and B cell subpopulation by flow cytometric analysis **(J)**. Hemagglutination inhibition (HI) antibody titers, viral shedding and cell proliferation assay results were analyzed with One-way ANOVA followed by a multiple comparisons Tukey's test. Survival curves were analyzed using the Long-Rank test. Statistical difference was considered with a *P<0*.*05*. Significant differences are denoted by different letters.

Very high concentrations of chIFNγ were detected in rZJ1*L/IFNγ-infected AF after treatment with BPL as well as in the untreated rZJ1*L/IFNγ-infected AF control (Fig 3A).

#### Chicken IFNγ bio-activity after treatment with BPL

Bio-activity of chIFNγ contained in the BPL-treated AFs used for inactivated vaccine preparation was confirmed by a macrophage activation assay consisting of the measurement of nitrites released to the cell culture supernatant as an indirect way to measure nitric oxide production from activated macrophages. BPL-rZJ1*L/IFNγ AF was able to induce nitrite production in HD11 cells, while very low levels of nitrites were detected in the BPL-AF and BPL-ZJ1*L treated cells (Fig 3B). These results together with the quantification of chIFNγ in BPL-rZJ1*L/IFNγ AF, confirmed that neither the concentration nor the bio-activity of chIFNγ were affected by treatment with BPL.

#### Effects of chIFNγ delivered through an inactivated vaccine system

The effects of chIFNγ delivered by the above inactivated vaccine virus were evaluated on the antibody-mediated immune response (AMI), the recall CMI response, and on virus shedding and survival after challenge. Evaluation of the AMI response through antibody titer determination by HI test demonstrated that the inactivated rZJ1*L/IFNγ, given at an EID_50_/mL of 10^8.1^, induced lower mean pre- (8.8) and post-boost (23.4) titers of antibodies specific to the vaccine virus rZJ1*L as compared to the control vaccines LS (37.4 and 92.85, respectively) and rZJ1*L (47.2 and 115.6, respectively) (Fig 3C). However when the vaccine dose was increased by one log (EID_50_/mL of 10^9.1^), the pre- and post-boost mean HI titers for the rZJ1*L/IFNγ-vaccinated group increased considerably (81.8 and 234.7, respectively) compared to the previous vaccine dose (Fig 3D). In addition, there was no significant change in the mean HI titer for neither the rZJ1*L-vaccinated group (72.5 and 177.8, respectively), nor for the LS-vaccinated group at this dose (38.2 and 69.4, respectively (Fig 3D). Additionally, the antibody response after challenge was also evaluated, but no statistical difference was found between rZJ1*L/IFNγ-, rZJ1*L- and LS-vaccinated groups, showing mean HI titers of 744.7, 810.7 and 907.6, respectively (Fig 3E). These results suggest that the rZJ1*L/IFNγ inactivated vaccine did not enhance the AMI response and that the chIFNγ, co-delivered with this vaccine system may have a negative effect on AMI response, depending on the vaccine dose. Similarly, the survival rate after challenge was negatively affected by the addition of chIFNγ, also without statistical significance. Approximately 92% of the birds vaccinated with rZJ1*L/IFNγ survived, while there was 100% survival for both LS- and rZJ1*L-vaccinated birds. By day 6 after challenge, 100% sham-vaccinated controls had succumbed to the virulent challenge (Fig 3F).

Inactivated rZJ1*L/IFNγ did not have a significant effect on viral shedding after challenge. All vaccinated groups numerically decreased oropharyngeal shedding compared to the sham-vaccinated control at 4dpc, however, the difference between sham-vaccinated and rZJ1*L groups was statistically significant. Moreover, there was no statistical difference between LS, rZJ1*L and rZJ1*L/IFNγ vaccinated-groups (Fig 3G). In addition, all vaccinated groups presented lower virus shedding in cloacal swabs, when compared to the sham-vaccinated control, but no difference was found between vaccinated groups at 2 or 4 dpc (Fig 3H). When the recall CMI response was evaluated, no significant differences on antigen-specific response were observed between groups (Fig 3I). Nonetheless, the rZJ1*L/IFNγ vaccinated-group showed numerically lower proliferation response to the antigen than the rZJ1*L-vaccinated group (Fig 3I). Lymphocyte populations from the spleens collected after booster vaccination were also monitored through flow cytometric analysis; however, no significant differences between groups were observed either (Fig 3J). These results showed once more that delivering chIFNγ together with vaccine antigen, did not enhance AMI, protection against mortality, or CMI response; in addition, it did not had an effect the amount of challenge virus shed. Collectively, the data suggest potential negative effects of co-delivering chIFNγ with NDV vaccine antigen.

## Discussion

Our studies demonstrate that the delivery of chIFNγ into chickens or embryos is achievable, however, the outcome of our vaccination experiments suggests that the process of modulating the immune response in order to achieve immune enhancement is likely to be highly complex. We have focused on the use of chIFNγ because the existing literature suggested that this cytokine should enhance the cellular and humoral immune responses to vaccines. For that effect we utilized three different ND vaccination systems to deliver chIFNγ, and studied the effect of co-delivering chIFNγ and NDV antigens on the immune response and protection upon challenge. These systems consisted of: 1) a DNA vaccine expressing NDV F gene co-administered *in ovo* with a plasmid expressing chIFNγ, 2) a recombinant live vaccine expressing chIFNγ administered *in ovo* and in 4-week-old SPF chickens, and 3) the same recombinant NDV vaccine expressing chIFNγ protein, used as an inactivated vaccine. While chIFNγ, was undoubtedly expressed and functionally active, surprisingly none of the three different delivery methods used enhanced the chicken immune response as measured by an increase in the humoral responses or by an improved survival rate after a virulent challenge.

A secondary conclusion of this study is that the use of chIFNγ could be detrimental to protection under certain delivery conditions. Co-delivery of chIFNγ via a DNA vaccine system had a negative effect on viral shedding after challenge. Those birds that received both the F and chIFNγ expression vectors shed significantly more challenge virus than the birds vaccinated only with vector expressing the F protein (Fig 1D), however, both groups secreted less viruses than the sham-vaccinated and pTriEX controls, thus indicating that the antigen was effectively delivered and was capable of inducing an immune response. Morbidity and mortality also increased significantly when chIFNγ was co-administered with the F gene (Fig 1E and 1F). The same negative trend was observed with the *in ovo* live vaccine system and the inactivated vaccine system. Both systems induced lower AMI response when chIFNγ was co-delivered with the vaccine antigen (Figs 2C and 3C). When comparing the results from the inactivated vaccine system shown in Fig 3C and 3D, the negative effect induced by chIFNγ was not visible with the higher vaccine dose. It is possible that the negative effect of chIFNγ may not be sufficiently pronounced to modify the immune response when a higher dose of vaccine was co-administered to the birds.

The difficulties on achieving a reliable enhancement of the immune response are revealed in the pre-existing literature. There have been a number of previous reports utilizing diverse systems to deliver chIFNγ. These reports have shown either positive or negative effects of the utilization of this cytokine as vaccine adjuvant to stimulate the immune response. Our results are in agreement with Sawant [32], Park [41], Schijns [42], Ding [36] and Min [37]. Sawant *et al*, Park *et al* and Schijns *et al* found no effect or negative effect on AMI response when chIFNγ was co-administered with the NDV, IBDV DNA or IBDV inactivated vaccine antigens, respectively [32, 41, 42]. In addition Sawant and Park also reported no effect on survival, or lower survival rates after challenge with vNDV or vvIBDV, respectively, compared to the control vaccine without cytokine [32, 41]. In contrast, results from the present research work conflict with numerous publications [31, 35, 38, 40, 42, 48]. As example, Yin and collaborators results showed increased survival after challenge (40%) and decreased challenge virus load from multiple organs and cloaca, when chIFNγ was co-administered. Surprisingly, after four applications of DNA NDV vaccines with plasmids expressing the NDV F and HN proteins conferred no protection against mortality (0% survival), while our DNA vaccine expressing only the F protein resulted in 83% survival of experimental subjects after two vaccine applications. Sawant found increased antigen-specific memory response [32]; and Binjawadagi *et al* and Lowenthal *et al* observed enhanced AIM [15, 35].

Because our studies focus only on the simultaneous delivery of antigen with chIFNγ the possibility of future improvement under different vaccination conditions cannot be discarded. Our data suggest that further studies should be conducted on determining proper timing and dose. Binjawadagi and collaborators reported increased NDV-specific antibody HI titers after vaccination and boost with live LaSota and R_2_B vaccines, with co-administration of chIFNγ either during vaccination or 6 hrs after vaccination [35]. The HI antibody titers increased when chIFNγ was co-administered during vaccination; however, the highest titers were induced when the cytokine was administered 6 hours after vaccination. While these studies reported increased HI titers with chIFNγ, those values were still below the protective limit (4 Log_2_) for NDV [35]. Lowenthal found that the intraperitoneal administration of 10 μg of purified chIFNγ did enhance the SRBC-specific antibody response [31]. Unfortunately, it is not possible to determine the levels of chIFNγ delivered in our DNA vaccines or live vaccine systems; however, we estimated (Fig 1A and data not shown) that our inactivated vaccine platform delivered approximately a hundred times less chIFNγ than Lowenthal’s, which may suggest that our inactivated vaccine system did not deliver enough cytokine to effectively enhance the AMI response. A third factor that may be important when using chIFNγ as a vaccine adjuvant may be the type of antigen employed. Ding *et al* and Min *et al* observed that when the cytokine was co-administered with DNA vaccines against 3-1E protein from *E*. *acervulina*, no effect or decreased body weight gain were observed along with reduction in number of oocysts shed [36, 37]; however, in another study, Xu and collaborators demonstrated that the co-administration of chIFNγ with a S07 (*E*. *tenella*) subunit vaccine increased body weight gain, antibody response and antigen-mediated lymphocyte proliferative response [40]. Additionally, Schijns *et al*. reported no significant effect of co-administration of chIFNγ together with an IBDV inactivated vaccine. However, when chIFNγ was co-administered with tetanus toxoid (TT) the TT-specific antibody titers increased at 2, 6, 9 and 12 weeks post immunization [42].

In conclusion, our results provide evidence that we were able to co-deliver NDV antigen and active chIFNγ through DNA, live or inactivated vaccine systems. However, none of these systems enhanced Newcastle disease virus vaccine immunogenicity or improved protection and survival following NDV vaccination and challenge. Collectively, our results, in combination with previously published work on the effects of chIFNγ as vaccine adjuvant, suggest that the immunomodulatory effect of chIFNγ may depend on other key factors, such as the relative time of application, the amount of cytokine delivered, and the type and amount of antigen co-delivered with the cytokine.
